# Decrease of PPAR**δ** in Type-1-Like Diabetic Rat for Higher Mortality after Spinal Cord Injury

**DOI:** 10.1155/2014/456386

**Published:** 2014-04-10

**Authors:** Cheng-Chia Tsai, Kung-Shing Lee, Sheng-Hsien Chen, Li-Jen Chen, Keng-Fan Liu, Juei-Tang Cheng

**Affiliations:** ^1^Department of Neurosurgery, Mackay Memorial Hospital, Taipei City 10449, Taiwan; ^2^Graduate Institute of Injury Prevention and Control, Taipei Medical University, Taipei City 10401, Taiwan; ^3^Department of Surgery, Kaohsiung Municipal Hsiao-Kang Hospital and Kaohsiung Medical University, Kaohsiung City 81201, Taiwan; ^4^Department of Obstetrics and Gynecology, Chi Mei Medical Center, Yong Kang, Tainan City 71101, Taiwan; ^5^Department of Medical Research, Chi Mei Medical Center, Yong Kang, Tainan City 71101, Taiwan; ^6^Department of Biotechnology, Southern Taiwan University, Yong Kang, Tainan City 71102, Taiwan; ^7^Institute of Basic Medical Sciences, College of Medicine, National Cheng Kung University, Tainan City 70101, Taiwan; ^8^Institute of Medical Science, College of Health Science, Chang Jung Christian University, Gui-Ren, Tainan City 71301, Taiwan

## Abstract

Changes in the peroxisome proliferator-activated receptors-**δ** (PPAR**δ**) expression in rats after spinal cord injury (SCI) have been previously reported. Diabetic animals show a higher mortality after SCI. However, the relationship between the progress of diabetes and PPAR**δ** in SCI remains unknown. In the present study, we used compressive SCI in streptozotocin-(STZ-) induced diabetic rats. GW0742, a PPAR**δ** agonist, was used to evaluate its merit in STZ rats after SCI. Changes in PPAR**δ** expression were detected by Western blot. Survival rates were also estimated. A lower expression of PPAR**δ** in spinal cords of STZ-diabetic rats was observed. In addition, the survival times in two-week induction diabetes were longer than those in eight-week induction group, which is consistent with the expression of PPAR**δ** in the spinal cord. Moreover, GW0742 significantly increased the survival time of STZ rats. Furthermore, their motor function and pain response were attenuated by GSK0660, a selective PPAR**δ** antagonist, but were enhanced by GW0742. In conclusion, the data suggest that higher mortality rate in STZ-diabetic rats with SCI is associated with the decrease of PPAR**δ** expression. Thus, change of PPAR**δ** expression with the progress of diabetes seems responsible for the higher mortality rate after SCI.

## 1. Introduction

Spinal cord injury (SCI) is defined as damage to the spinal structure and function that can be caused by a host of etiological factors, including labor injuries and traffic accidents; the condition also creates enormous physical and emotional cost to individuals [1]. SCI is easily led to motor paralysis and sensory dysfunction while both afferent sensory and efferent motor innervations are passed through spinal cord [2]. The sensory dysfunction is associated with urinary impairment, which is a major factor in morbidity and even mortality in those with SCI [3]. Diabetes mellitus (DM) is a metabolic disorder with many chronic complications, and diabetic patients are more vulnerable to traumatic injury [4]. STZ-diabetic rats provide a helpful animal model as type-1-like DM to investigate the correlation between diabetes and SCI [5], while the identifying of an agent to address the specific needs of diabetic patients with SCI is urgent.

Peroxisome proliferator-activated receptors (PPARs) are ligand-activated transcription factors belonging to the nuclear hormone receptor superfamily, which includes the classical steroid, thyroid, and retinoid hormone receptors [6]. At present, three PPAR subtypes have been identified and are commonly designated as PPAR*α*, PPAR*δ*, and PPAR*γ* [7]. Some reports have shown that PPARs are involved in the pathogenesis of several diseases, including diabetes mellitus, obesity, atherosclerosis, neurological diseases, and SCI [8–10].

It has been documented that GW0742 (a selective agonist of PPAR*δ*) can reduce the development of inflammation and tissue injury associated with SCI [11]. Additionally, specific antagonists or blockers have been applied to elucidate the potential action mechanism(s) of PPAR*δ*. The present study is designed to investigate the role of PPAR*δ* levels in the spinal cord of type-1-like diabetic rats induced by streptozotocin (STZ-diabetic rats) in the mortality after SCI and to determine the effects of GW0742 on SCI in STZ-diabetic rats.

## 2. Materials and Methods

### 2.1. Experimental Animals

The male Wistar rats obtained from the Animal Center of the National Cheng Kung University Medical College were maintained in a temperature-controlled room (25 ± 1°C) under a 12 h light-dark cycle (lights on at 06:00). All rats received water and standard chow (Purina Mills, LLC, St. Louis, MO, USA)* ad libitum*. All animal-handling procedures were performed according to the Guide for the Care and Use of Laboratory Animals of the National Institutes of Health and followed the guidelines of the Animal Welfare Act.

### 2.2. Induction of STZ-Diabetic Rats

Male diabetic rats were induced using an intravenous injection (i.v.) of streptozotocin (STZ; Sigma Chemical Co., St. Louis, MO) (65 mg/kg) into Wistar rats. Animals were considered to be diabetic if the plasma glucose reached 280 mg/mL or greater, in addition to the presence of polyuria and other diabetic signs, as described previously [12]. In the present study, we used STZ-diabetic rats after a two-week induction (2W-STZ) period or after an eight-week induction (8W-STZ) period for comparison.

### 2.3. Spinal Cord Injury (SCI)

The spinal cord injury (SCI) was performed mainly according to our previous method [13] with some modifications [14]. In brief, the laminectomy for removal of the vertebral peduncle was performed between T8 and T9 on rats under anesthesia with sodium pentobarbital (30 mg/kg, intraperitoneally; Sigma Chemical Co., St. Louis, MO). We used a calibrated aneurysm clip with a closing pressure of 55 g to place between the dorsal and ventral surfaces of spinal cord for 1 min. Animals that received the same laminectomy without compression with clip were grouped as the sham-operated control. Then, we treated all animals with 0.1 mL of cefazolin injection (10 mg/kg body weight; China Chemical Pharmaceutical Co., Ltd., Taipei, Taiwan) for 3 days after the surgery. Animals that received SCI were individually housed on special bedding to prevent the formation of pressure sores. Additionally, rats had their bowels and bladders manually compressed twice daily. Food and water were supplied at a lowered height in their cages and were freely accessible [13]. Similar to the previous report [13], the rats typically did not survive beyond 4-5 weeks after the SCI. In the present study, GW0742 (a selective agonist of PPAR*δ*) and GSK0660 (a selective antagonist of PPAR*δ*) purchased from Tocris Bioscience (Ellisville, MO, USA) were dissolved in DMSO and diluted with saline. The solution of GW0742 or GSK0660 was intravenously injected into the rats via tail vein. Additionally, we employed the vehicle at the same volume to treat the control group in the same manner.

### 2.4. Survival Protocol

We determined the survival rate in rats after SCI. All rats were divided into four groups: normal Wistar, 2W-STZ, and 8W-STZ treated with/without GW0742. They were housed in a clean and dry room at 20–26°C; standard chow and water were freely available 12 h later. Mortality was followed and checked every day for 22 days after SCI.

### 2.5. Locomotor Scale

According to previous report [15], the Basso, Beattie, and Bresnahan (BBB) locomotor rating scale (locomotor scale) from 0 to 21, where zero reflects no locomotor function and 21 reflects normal performance, is used to evaluate the effects of GW0742 (0.3 mg/kg) or GSK0660 (0.1 mg/kg) on functional recovery after SCI. We arranged the rats to walk around freely in a 90 cm^2^ field (width and length) for 4 minutes and movements of the hindlimb were observed continuously. Rats were trained to gently adapt in the field at first. Two investigators conducted 4 min testing sessions on each leg of the rats walking continuously in the field. This study started one day after injury and continued for 20 days or over. The functional deficits were double blind checked by the trained investigators. The results of behavior outcomes and examples of locomotor scores were also recorded in the digital video.

### 2.6. Inclined Plane Test

We applied the inclined plane test (IPT) to evaluate the ability of rats to maintain their position for 5 s on an inclined plane that was covered with a rubber mat containing horizontal ridges (1 mm deep, spaced 3 mm apart, and self-made), as described previously [16]. The rats were determined as the angle of the surface was increased from 5 to 90° at 5° intervals. The angle at which the rat could not maintain its position was the outcome measured.

### 2.7. Limb Hanging Test

This test is widely used to evaluate both forelimb and hindlimb function. However, as mentioned in a previous report [17], it is mainly employed to test muscle function in the forelimbs of animals that received SCI. The test is conducted using a 12 cm long and 1.8 mm wide rounded metal rod applied to the volar surface of the forepaw to record the presence or absence of grasping and the release time in seconds. As the rod is elevated above the surface and suspended, characterization of the animal's forelimb muscle strength is possible. In addition, contact of the body, hindlimb, or tail with the ground or parts of the equipment on the sides should be prevented. The time for rat suspended on the rod is measured. Following the previous method [17], this test was typically repeated five times and the mean value was then calculated.

### 2.8. Pain Test

After training to stay in test chambers, rats were divided to sham and SCI groups randomly. Similar to the previous method [18], the response of foot withdrawal after each mechanical notching was determined by a flat-tipped cylindrical probe to measure 200 *μ*m in diameter. Each stimulus was performed about one second under the interval approximately 10 to 15 seconds at the force shown in newtons (N). The incidence of positive response was estimated for comparison in two groups.

### 2.9. Western Blotting Analysis

Spinal cord tissues were isolated from rat with 2-week or 8-week induction of diabetes. Also, another set is isolated from STZ-diabetic rats on the 7th, 14th, or 21st day after SCI. The isolated tissues were homogenized in the ice-cold buffer solution containing 10 mM Tris-HCl (pH 7.4), 20 mM EDTA, 10 mM EGTA, 20 mM *β*-glycerophosphate, 50 mM NaF, 50 mM sodium pyrophosphate, 1 mM phenylmethylsulfonyl fluoride, and the protease inhibitors 25 *μ*g/mL leupeptin and 25 *μ*g/mL aprotinin. The mixture was then centrifuged at 1000 ×g for 10 min. The obtained supernatant was further centrifuged at 48,000 ×g for 30 min. After resuspension of the pellet in ice-cold Triton X-100 lysis buffer, samples were then centrifuged at 14,010 ×g for 20 min. The above centrifugations all performed at 4°C. The supernatant was collected in Eppendorf tube to store at −80°C. The membrane extracts (20–80 *μ*g) in supernatant were applied for separation using 10% SDS-polyacrylamide gel electrophoresis. The obtained proteins were transferred onto a BioTraceTM polyvinylidene fluoride (PVDF) membrane (Pall Corporation, Pensacola, FL, USA) for 2 hours. The blots were developed through the reaction with primary antibodies of PPAR*δ* (Abcam, Cambridge, UK) for 16 hours. Then, they were hybridized with horseradish peroxidase-conjugated rabbit anti-rabbit IgG (Jackson ImmunoResearch Laboratories, Inc., PA, USA) for 2 hours and developed with the Western Lightning Chemiluminescence Reagent PLUS (PerkinElmer Life Sciences Inc., Boston, MA, USA). We employed Gel-Pro Analyzer software 4.0 (Media Cybernetics, Silver Spring, MD, USA) to quantify the densities of obtained immunoblots at 40 KDa for PPAR*δ* and 43 KDa for actin, respectively.

### 2.10. Statistical Analysis

All results were expressed as the mean ± SE of each group. Statistical analysis was performed using ANOVA analysis with the Newman-Keuls post-hoc ANOVA. After the calculation of survival using the Kaplan-Meier estimate, the log-rank test and the Chi-squared test were used to compare the survival curves in two groups. A *P* value of 0.05 or less was considered statistically significant.

## 3. Results

### 3.1. Effects of SCI on Survival in STZ-Diabetic Rats

**Figure 1:**
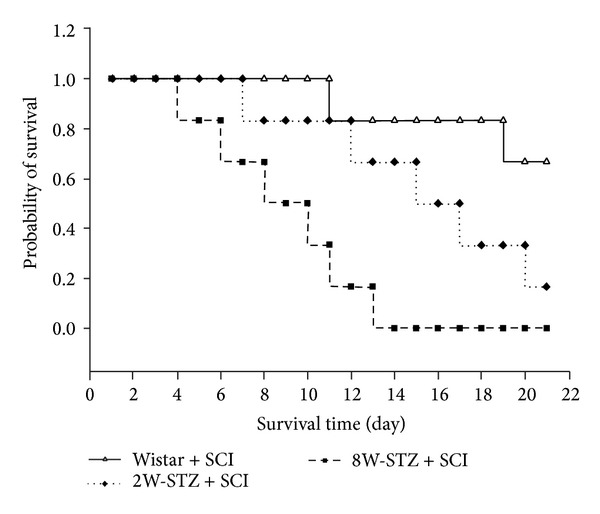
The effect of SCI on survival ability in STZ-diabetic rats. STZ-diabetic rats were obtained from the two-week induction group (2W-STZ) and the eight-week induction group (8W-STZ). Data represent the survival rate of ten animals in each group.

After SCI, the survival days in normal rats were longer than in STZ-diabetic rats. The survival time in normal rats with SCI had a mean of 35 days, while the eight-week induction STZ-diabetic rats with SCI lasted a mean of 13 days, which indicated a marked difference in survival time between two groups. In addition, we compared the maximum survival time in diabetic rats after SCI. As shown in Figure 1, the eight-week induction STZ-diabetic rats showed a significantly higher mortality than the two-week induction STZ-diabetic rats (*P* < 0.001).

### 3.2. Effects of PPAR*δ* on Mortality in STZ-Diabetic Rats with SCI

**Figure 2:**
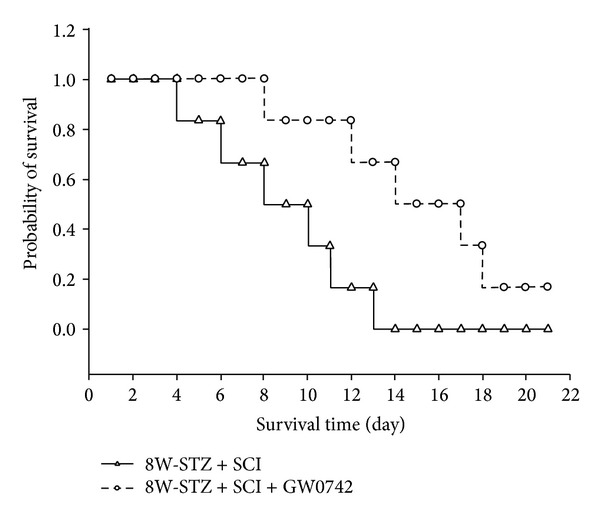
The effect of PPAR*δ* activation on survival ability in STZ-diabetic rats after SCI. The eight-week induction group (8W-STZ) was intravenously injected with GW0742 (0.3 mg/kg, once daily). Data represent the survival of ten animals.

Intravenous injection of GW0742 (0.3 mg/kg, once daily) [19] markedly increased the survival period after SCI in the eight-week induction STZ rats (Figure 2). The survival time in STZ-diabetic rats with SCI that received GW0742 was 20 days while the STZ-diabetic rats with SCI that received vehicle lasted only 13 days, suggesting significant beneficial effect of PPAR*δ* on survival time in STZ-diabetic rats (*P* < 0.001). Also, animals were followed continuously to determine the maximal survival time until all animals died.

### 3.3. Effects of PPAR*δ* Agonist/Antagonist on Motor Function and Pain Response in STZ-Diabetic Rats with SCI

**Figure 3:**
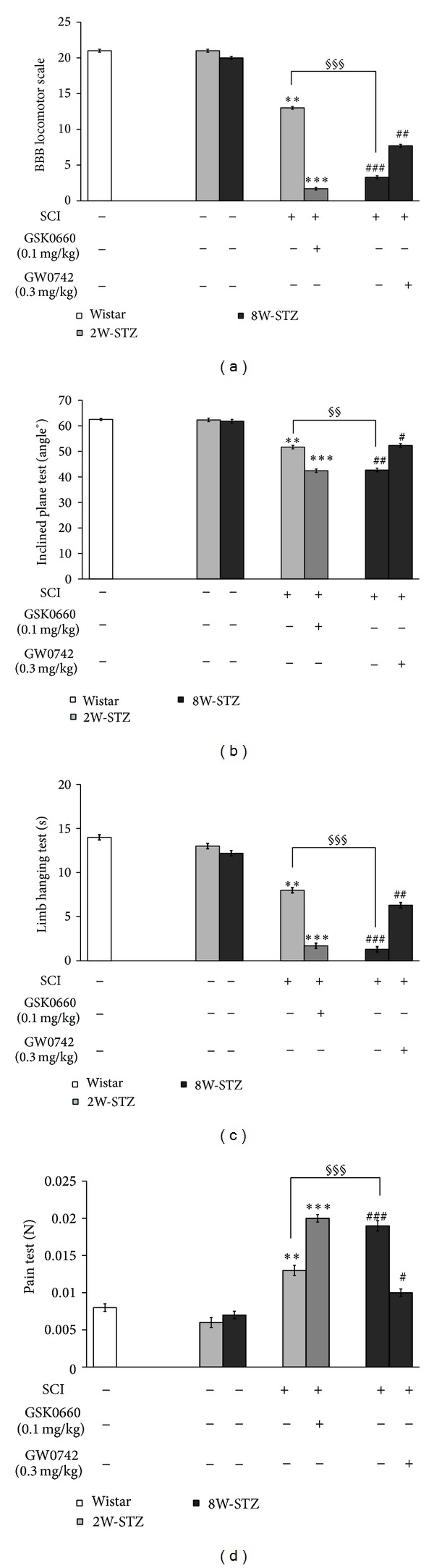
Changes in behavioral and pain tests in diabetic rats after STZ induction for two weeks (2W-STZ) or eight weeks (8W-STZ) receiving SCI surgery or sham operation. The 2W-STZ receiving SCI was further treated with/without GSK0660 (0.1 mg/kg) intravenously once daily for 21 days while the 8W-STZ receiving SCI was further treated with/without GW0742 (0.3 mg/kg) intravenously once daily for 14 days. (a) BBB locomotor scale, (b) inclined plane test, (c) limb hanging test, and (d) pain test. Values (mean ± SE) were obtained from each group of six rats. **P < 0.01 and ***P < 0.001 compared with the 2W-STZ group. ^#^ *P* < 0.05, ^##^ *P* < 0.01, and ^###^ *P* < 0.001 compared with the 8W-STZ group. ^§§^ *P* < 0.01 and ^§§§^ *P* < 0.001 compared with the 2W-STZ + SCI group.

As shown in Figure 3, the group of 8W-STZ showed a significant difference from 2W-STZ including data of the BBB locomotor scale, inclined plane test, limb hanging test, and pain test (*P* < 0.01 and *P* < 0.001). Additionally, intravenous injection of GSK0660 (0.1 mg/kg, once daily) to 2W-STZ as described previously [20] for 21 days further attenuated the motor functions and pain responses in comparison to untreated 2W-STZ. Furthermore, intravenous injection of GW0742 (0.3 mg/kg, once daily) to 8W-STZ for 14 days improved the motor functions and pain responses in comparison to untreated 8W-STZ.

### 3.4. Changes in PPAR*δ* Expression in STZ-Diabetic Rats

**Figure 4:**
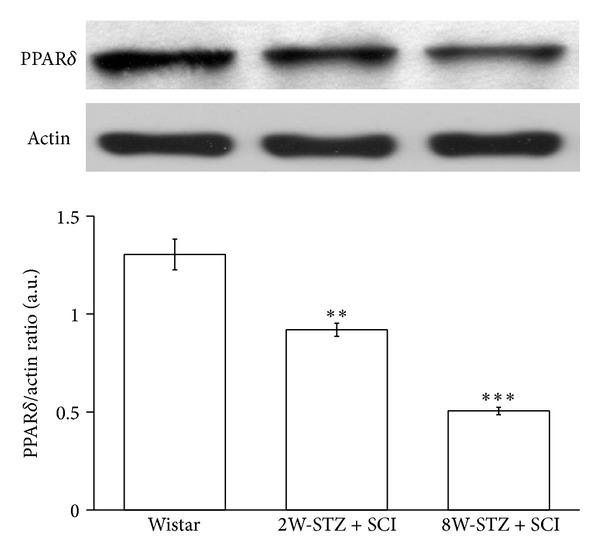
The expression of PPAR*δ* in the spinal cord obtained from normal or STZ-diabetic rats. The spinal cords of diabetic rats were obtained from the two-week induction group and the eight-week induction group. Data represent the mean ± SEM of six animals. **P < 0.01 and ***P < 0.001 compared with the normal group.

After evaluating the behavioral tests, we used the spinal cord from each rat in the same group to perform the Western blotting analysis. As shown in Figure 4, the PPAR*δ* expression in the spinal cords of STZ-diabetic rats was markedly lower than in normal rats (*P* < 0.01). Additionally, the PPAR*δ* expression in the eight-week induction STZ-diabetic rats was much lower than that in the two-week induction group (*P* < 0.001).

### 3.5. The Effects of SCI on PPAR*δ* Expression in STZ-Diabetic Rats

**Figure 5:**
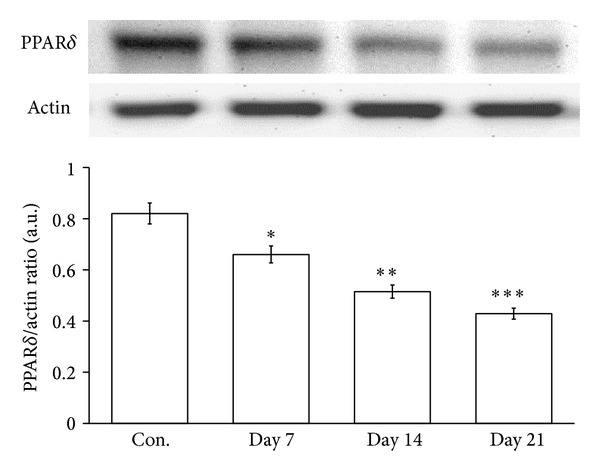
Changes in PPAR*δ* expression in the spinal cord of STZ-diabetic rats after SCI. PPAR*δ* expression in the spinal cord of the two-week induction STZ-diabetic rats was investigated on the 7th, 14th, and 21st days after SCI. Data represent the mean ± SEM of six animals. *P < 0.05, **P < 0.01, and ***P < 0.001 compared with the sham-operated group (Con.).

As shown in Figure 5, we compared the PPAR*δ* expression on the 7th, 14th, and 21st days after SCI in the two-week induction STZ rats. The results showed that PPAR*δ* expression was reduced after SCI in STZ-diabetic rats in a time-dependent manner.

## 4. Discussion

In the present study, we found that STZ-diabetic rats have a higher mortality rate than normal rats after SCI. A decrease in PPAR*δ* expression was observed in spinal cords of STZ-diabetic rats, and this change was more marked with the progress of diabetes. In addition, the survival period of STZ-diabetic rats after SCI was markedly increased by GW0742 at a dose sufficient to activate PPAR*δ* [11]. Also, the motor dysfunction and pain responses were improved in 8-week induced STZ-diabetic rats (8W-STZ) treated with GW0742 after SCI. Furthermore, the motor dysfunction and pain responses became more marked in 2-week induced STZ-diabetic rats (2W-STZ) with SCI after treatment with GSK0660 at the dose effective to block PPAR*δ* [11]. Thus, change of PPAR*δ* expression seems associated with the progress of diabetes and the higher mortality rate after SCI.

Primary traumatic mechanical injury in the spinal cord causes damage to neurons, which cannot be recovered or regenerated. Thus, animals after SCI showed a short survival time. Following a previous report [13], rats survived approximately 4-5 weeks after compression SCI and we determined the survival rate at similar days. However, some studies showed a longer survival time (about 11 weeks) in rats with SCI [21, 22]. Actually, rats with SCI showed 73% of survival rate in the period of this study. Thus, the total survival time will be the same as we determined it for a longer time. Interestingly, higher mortality rate was observed in diabetic rats with SCI and the mortality was more marked in 8W-STZ than in 2W-STZ. This supports our hypothesis that diabetes may produce higher mortality rate in rats with SCI.

Recently, it has been suggested that the pharmacological activation of PPAR*δ* can be considered a potential target because of its anti-inflammatory/antioxidant/antiexcitotoxic/proenergetic profile in some neurological and inflammatory-related diseases [23]. PPAR*δ* has drawn much attention as a drug discovery target for regulating glucose and lipid metabolism [24] because PPARs are widely distributed throughout the body and are mainly known for their effects on metabolism. Three isoforms of PPAR have been identified: PPAR*α*, PPAR*δ* (also called PPAR*β*), and PPAR*γ* [25]. PPAR*α* and PPAR*γ* are involved in the metabolism of lipid and glucose. Basically, PPAR*δ* is the most abundant PPAR isoform in the body and many studies have demonstrated its role in antioxidative stress [26] and neuroprotection [27]. Moreover, diabetes attenuated the recovery and increased mortality after SCI, which was related to a reduced ability to repair the injured tissue and to recover the neurological function [28]. Similar to our results (Figures 1 and 3), STZ-diabetic rats showed higher mortality than normal rats after SCI.

In the central nervous system, PPAR*δ* is expressed mainly in oligodendrocytes and neurons [29]. GW0742 reduced the cellular and molecular changes occurring in SCI by targeting different downstream pathways, thereby modulating PPAR*δ* receptors [30]. It has been indicated that GW0742 treatment ameliorates the tissue injury associated with SCI in Wistar rats through increased PPAR*δ* expression and this action of GW0742 was blocked by GSK0660 [11]. Thus, the changes of PPAR*δ* expression in Wistar rats were not investigated in the present study. This may interrupt the understanding of difference between SCI in normal and diabetic rats. However, the main aim of this report is to characterize the role of PPAR*δ* with diabetic progress in higher mortality of rats with SCI.

To evaluate the effects of PPAR*δ* on motor function and pain responses in STZ-diabetic rats after SCI, several behavioral tests were applied, including the BBB locomotor scale, inclined plane test, and limb hanging test [31–33]. The motor functions of diabetic rats can be influenced after SCI by the pharmacological manipulation of PPAR*δ* activity, as described previously [11]. As shown in Figure 3, we found that the blockade of PPAR*δ* by GSK0660 enhanced motor dysfunction and pain insensitivity. In contrast, the activation of PPAR*δ* by GW0742 improved motor dysfunction and increased pain sensitivity (Figure 3). Thus, PPAR*δ* plays an important role in the recovery from SCI in STZ-diabetic rats, and this role has been identified using behavioral tests. It has also been shown that PPAR*δ* is highly expressed in the central nervous system [34], with a great influence on neuronal cell function [35]. In the present study, PPAR*δ* expression in the spinal cord was compared between normal and STZ-diabetic rats to indicate that diabetes reduced PPAR*δ* expression in the spinal cords of rats (Figure 4) and is associated with STZ-induced hyperglycemia and systemic inflammation [36, 37]. Moreover, we further investigated changes in PPAR*δ* expression after SCI in STZ-diabetic rats. As shown in Figure 5, PPAR*δ* expression in the spinal cords of STZ-diabetic rats decreased in a time-dependent manner after SCI; this pattern is similar to previous reports in normal rats [38, 39]. Our data showed that the STZ-diabetic rats lack enough PPAR*δ* sufficient to repair the damaged neurons. The SCI injury was worse in diabetic rats, mainly due to the decreased PPAR*δ* in their spinal cords. Thus, the activation of PPAR*δ* is helpful to improve the injury from SCI in STZ-diabetic rats. However, more experiments for understanding the potential mechanism(s) of PPAR*δ* in STZ-diabetic rats with SCI are required in the future.

## 5. Conclusions

We found that PPAR*δ* is lowered in the spinal cords of STZ-diabetic rats and that it can be further reduced by SCI. This finding is helpful for explaining the higher mortality in STZ-diabetic rats after SCI. Thus, PPAR*δ* provides a novel target for the development of therapeutic agents in the treatment of diabetic patients after SCI.
